# Influence of hemodialysis on circulating CD4^low^CD25^high^ regulatory T cells in end-stage renal disease patients

**DOI:** 10.1007/s00011-013-0679-z

**Published:** 2013-11-05

**Authors:** Katarzyna A. Lisowska, Alicja Dębska-Ślizień, Aleksandra Jasiulewicz, Ewa Bryl, Jacek M. Witkowski

**Affiliations:** 1 Department of Pathophysiology, Medical University of Gdańsk, Gdańsk, Poland; 2 Department of Nephrology, Transplantology and Internal Diseases, Medical University of Gdańsk, Gdańsk, Poland; 3 Department of Pathology and Experimental Rheumatology, Medical University of Gdańsk, Gdańsk, Poland

**Keywords:** T regulatory cells, Inflammation, End-stage renal disease, Hemodialysis

## Abstract

**Objective:**

Immunodeficiency of end-stage renal disease (ESRD) is caused by several factors including uremic toxins and biocompatibility reactions due to the repeated hemodialysis (HD) procedure. It has also been suggested that poor T cell responses could be associated with the increased number of regulatory T cells (Tregs) which are necessary to limit the function of activated T cells. The aim of the study was to determine the proportion of CD4^+^CD25^+^ cells (activated T cells) to CD4^low^CD25^high^ cells (Tregs) within the CD4^+^ population in ESRD patients.

**Patients and methods:**

Two groups of ESRD patients, predialysis patients treated conservatively and patients undergoing hemodialysis (HD), as well as healthy controls were included in the study. Percentages of activated and regulatory T cells were determined ex vivo with flow cytometry based on the expression of CD4 and CD25 antigens.

**Results and conclusions:**

HD patients showed an increased percentage of CD4^+^CD25^+^ cells when compared with healthy controls, while there was no difference in the percentage of CD4^low^CD25^high^ cells between the patient groups. In our opinion, the repeated hemodialysis procedure significantly disturbs the balance between activated T cells and regulatory T cells in ESRD patients. Lack of Treg mobilization and chronic stimulation of T cells may contribute to the immune deficiency observed in these patients.

## Introduction

Despite developments in end-stage renal disease (ESRD) therapy, nearly half of hemodialysis (HD) patients suffer from chronic inflammation which is caused by several factors including uremic toxins and biocompatibility reactions [1]. It causes a chronic stimulation of lymphocytes which results in immunodeficiency manifested by susceptibility to infections (reviewed in [2]) and poor response to vaccination [3]. In vitro, CD4^+^ T cells of HD patients present decreased proliferative capacity as well as decreased interleukin 2 (IL-2) and interferon gamma (IFN-γ) production in response to stimulation [4–6]. However, it has also been suggested that poor T cell responses could be associated with the increased number or inappropriate function of regulatory T cells (Tregs) which are necessary to limit the function of activated T cells. Tregs as the master regulators of immune response act either by secreted cytokines or cell-to-cell interactions with effector T cells (reviewed in [7]). There is still little known about the role of Tregs in the development of immunodeficiency in ESRD. Moreover, contrasting results have been reported. Hendrikx et al. [8] observed a lower number and an impaired function of CD4^+^CD25^high^ regulatory T cells. In response to the article of Hendrikx et al., Libetta et al. [9] reported a significantly higher number and percentage of CD4^+^CD25^high^ cells in HD patients. According to Meier et al. [10], high levels of low-density lipoproteins in HD patients reduces the number of Tregs and interferes with their function.

Identification of regulatory T cells is still problematic, especially after discovering that transcription factor FoxP3 (forkhead box P3), currently the most reliable marker for Tregs, is also induced upon activation of CD4^+^ T cells [11]. According to Bryl et al. [12], significantly low expression of CD4 simultaneous with high expression of CD25 is a sufficient marker of functional human Tregs. Cells with this phenotype express perforin and granzyme B which allows them to kill effector cells directly [12]. Therefore, we used this phenotypic criterion to analyze proportions of CD4^+^CD25^−^ cells (naive T cells), CD4^+^CD25^+^ cells (activated T cells) and CD4^low^CD25^high^ cells (Tregs) within populations of CD4^+^ cells in ESRD patients, those not yet treated with dialysis (predialysis, PD) and those already hemodialyzed (HD), as well as in healthy controls.

## Patients and methods

### Patients

The inclusion criteria for patients was the fifth stage of chronic kidney disease (CKD). The study group consisted of 19 ESRD patients and 10 healthy volunteers. Nine of the patients were undergoing 5-h sessions of hemodialysis three times a week (the average length of time on hemodialysis was 13.83 ± 20.32 months). The remaining ten patients were being treated conservatively (predialysis, PD). The exclusion criteria for patients and healthy volunteers included infections, inflammation, malnutrition, neoplasm and blood loss during the study. None of the patients received erythropoiesis stimulating agents (ESA). The study was approved by the Ethical Committee of the Medical University of Gdańsk.

### Flow cytometry

Five milliliters of venous peripheral blood from patients and healthy volunteers was collected in EDTA-coated tubes. Samples of 50 μl blood per tube were transferred for staining with monoclonal antibodies and red blood cell (RBC) lysis as previously described [4]. Blood cells were incubated with fluorescein isothiocyanate (FITC)-conjugated anti-CD3, R-phycoerythrin (RPE)-Cy5-conjugated anti-CD4 (DAKO, Denmark) and PE-conjugated anti-CD25 (BD-Pharmigen, USA) for 30 min at 4 °C and analyzed on FACScan (Becton–Dickinson, USA).

### Statistical analysis

**Fig. 1:**
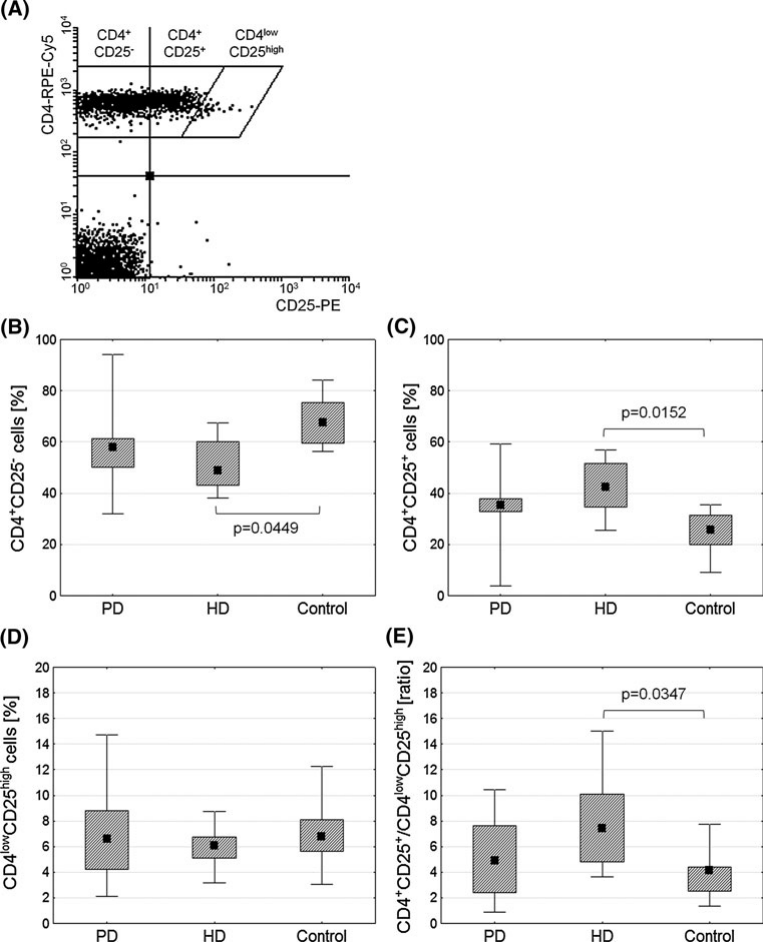
Comparison of activated and regulatory T cells within the CD4^+^ population ex vivo. T regulatory cells were identified based on low expression of CD4 simultaneous with high expression of CD25 (**a**) as described by Bryl et al. [12]. *Graphs* demonstrate percentages of CD4^+^CD25^−^ (**b**), CD4^+^CD25^+^ (**c**), CD4^low^CD25^high^ cells (**d**), and CD4^+^CD25^+^/CD4^low^CD25^high^ ratio (**e**) in predialysis (PD) and hemodialyzed (HD) patients and healthy controls. Midpoints of figures represent medians, boxes represent 25–75 % and whiskers outside represent the minimum and maximum of all the data, Kruskal–Wallis and post hoc test, *p* < 0.05

T cells were selected on the basis of their forward and side scatter characteristic and CD3 expression with Cyflogic, version 1.2.1 (©Perttu Terho and ©CyFlow Ltd). T regulatory cells were identified based on their low CD4 expression simultaneous with high CD25 expression (Fig. 1a) as described by Bryl et al. [12]. In order to distinguish CD4^low^CD25^high^ (regulatory T cells) from CD4^+^CD25^+^ cells (activated T cells), we applied the rhomboid regions to avoid overlapping of the lowest CD25 signal level shown by the CD4^low^ population and the highest one shown by other CD4^+^ cells [12].

Statistical analysis was performed using the Statistica, version 8 (StatSoft, Poland). The significance tests were chosen according to data distribution with the level of significance *p* ≤ 0.05.

## Results

The main clinical and immunological features of patients and controls are presented in Table 1. All groups were similar regarding age and gender. Creatinine was significantly increased while hemoglobin and RBC were significantly decreased in PD and HD patients when compared with healthy controls. The percentage of lymphocytes was also significantly decreased in PD patients when compared with healthy controls. The percentage and absolute number of CD4^+^ cells did not differ between the groups. There was also no difference in the number of CD4^+^ subpopulations with a different expression of CD25 antigen (Table 1).

**Table 1:** Clinical and immunological parameters in patients and healthy controls

	PD patients	HD patients	Controls	*p* value
Number	10	9	10	
Sex (M/F)	7/3	8/1	7/3	
Age	62.50 (23, 80)	65 (29, 81)	52.50 (31, 72)	0.4906
GFR (min/ml/1.73 m^2^)	11.26 (6.40, 22)	8.37 (3.49, 13)	>60	0.2672
Creatinine (mg/dl)	4.80 (1.88, 7.34)	5.90 (5.60, 13.57)	0.79 (0.65, 1.18)	**0.0019**
Hemoglobin (g/dl)	10.31 (9.10, 13.10)	10.75 (9.58, 12.30)	14.28 (12.60, 14.90)	**0.0002**
RBC (T/L)	3.51 (2.56, 4.44)	3.54 (3.13, 3.92)	4.55 (3.36, 5.02)	**0.0023**
WBC (G/L)	6.59 (4.99, 15.14)	5.87 (4.56, 6.20)	5.67 (3.81, 8.86)	0.2519
Lymphocytes (G/L)	1.10 (0.64, 2.18)	1.45 (0.83, 2.40)	1.65 (0.94, 2.46)	0.1020
Lymphocytes (%)	16.17 (7.20, 30.50)	25.05 (14.26, 38)	29.30 (17.85, 36.90)	**0.0159**
CD4^+^ cells (%)	38.52 (16.88, 53.44)	45.56 (18.52, 65.45)	38.19 (26.11, 47.07)	0.2674
CD4^+^ cells (G/L)	2.29 (1.19, 7.47)	2.49 (1, 3.14)	2.29 (1.03, 4.16)	0.7827
CD4^+^CD25^−^ cells in CD4^+^ cells (G/L)	3.76 (2.03, 13.33)	2.63 (1.79, 4.22)	3.98 (2.49, 5.77)	0.1491
CD4^+^CD25^+^ cells in CD4^+^ cells (G/L)	2.41 (0.21, 5.28)	2.55 (1.49, 3.27)	1.63 (0.51, 2.88)	0.1031
CD4^low^CD25^high^ cells in CD4^+^ cells (G/L)	0.51 (0.11, 0.96)	0.37 (0.17, 0.52)	0.40 (0.19, 0.71)	0.4850

Values are given as median (min, max), Kruskal–Wallis and post hoc test, *p* < 0.05; Mann–Whitney *U* test was used to compare GFR between HD and PD patients, *p* < 0.05. Significant differences are highlighted in bold

*GFR* glomerular filtration rate, *RBC* red blood cells, *WBC* white blood cells, *PD* predialysis, *HD* hemodialysis

Figure 1 shows the proportion of CD4^+^CD25^−^, CD4^+^CD25^+^ and CD4^low^CD25^high^ cells within the CD4^+^ population in ESRD patients and healthy controls. The percentage of CD4^+^CD25^−^ cells in HD patients was significantly decreased compared with healthy controls (Fig. 1b) while the percentage of CD4^+^CD25^+^ cells was significantly increased (Fig. 1c). No difference was seen in the percentage of CD4^low^CD25^high^ cells in the examined groups (Fig. 1d). There was an increase in the ratio of CD4^+^CD25^+^ to CD4^low^CD25^high^ in HD patients (Fig. 1e).

## Discussion

The balance between activated T cells and Tregs is crucial for immune homeostasis. This process involves activation of T cells followed by the expansion of Tregs in order to limit the function of activated T cells. It has been suggested that deficient responses of T cells in ESRD patients could be partly associated with the increased number of circulating Treg cells. However, articles on Tregs present conflicting results when it comes to the proportions of CD4^+^CD25^high^ regulatory T cells in these patients [8–10, 13].

The immune system of ESRD patients is affected by many factors including uremic toxins, bacterial products, repeated infections and biocompatibility reactions [1]. As a result, there is an activation of T cells seen as disturbances in subpopulations of cells ex vivo; HD patients are characterized by a reduced percentage of CD4^+^ cells with CD69 antigen, an early marker of activation, and an increased percentage of cells with late activation markers, like CD95 and HLA-DR [14]. The consequence of premature activation of T cells is their impaired proliferative capacity and decreased cytokine production [4–6, 14]. Our current results show an imbalance between CD4^+^CD25^+^ (activated T cells) and CD4^low^CD25^high^ (Tregs) in HD patients which is the result of an increase in the percentage of the former. This demonstrates the absence of regulatory T cell mobilization despite the presence of T cell activation in these patients. Recently Afzali et al. [13] also showed that there is no difference in the percentage and absolute number of CD4^low^CD25^high^ cells between HD patients and healthy controls which is consistent with our results. However, Afzali et al. reported that Tregs of HD patients were less suppressive and produced IL-17. Insufficient Treg activity is important in the context of transplantation; however, rapamycin-based protocols to induce tolerance to renal allografts could provide a solution to this problem since in vitro studies have shown that rapamycin promotes expansion of Tregs [13, 15]. Libetta et al. [9], who reported significantly larger numbers of CD4^+^CD25^high^ cells in HD patients, suggested that the use of different membranes could play a role in Treg mobilization; a low biocompatibility membrane would activate regulatory T cells.

Variations in reports on the percentage or the number of circulating regulatory T cells probably result from the use of different Treg markers and different methods of analysis. It is also worth noting that researchers rarely examine the ratio of activated to regulatory T cells, which is a useful factor in evaluating whether there is a balance between these cells. Our results show that the proportions of activated and regulatory T cells may be affected in the various groups studied, despite the lack of differences in the number of cells of a given population. Nevertheless, it appears that hemodialysis not only contributes to the excessive activation of T cells, but also interferes with the mobilization of Tregs, thereby disturbing the balance between these cells in ESRD patients.
